# Health and work: new times, new paradigms

**DOI:** 10.1590/0102-311XEN176323

**Published:** 2024-02-19

**Authors:** Ricardo Cordeiro, Ada Avila Assunção

**Affiliations:** 1 Faculdade de Ciências Médicas, Universidade Estadual de Campinas, Campinas, Brasil.; 2 Universidade Federal de Minas Gerais, Belo Horizonte, Brasil.

Is traditional knowledge able to address the current relationships between health and work? The perspective that aligned the ideas aimed to encourage the willingness of researchers, professionals, and service technicians to face the limits of theoretical and methodological frameworks adopted so far due to changes related to the globalized economy. Technological innovations in old and new production processes, loss of labor and social security rights that were once guaranteed, and weakening of trade union organizations have changed labor relations. The characteristics of employment, as discussed later in this article, have generated insecurity for both employed individuals or people looking for a job ^1^ ^,^ ^2^. This article has two focal points: criticize old ways of conducting research that are insufficient nowadays and provide suggestions for current research and investigations addressing new problems.

In the 1970s, at least two factors contributed to the onset of another crisis of capitalism: a drop in productivity achieved in the previous period of uninterrupted growth and inflationary process associated with the oil crisis in 1973, among other issues. In this context, the dispute for markets found obstacles in the series production and standard employment characteristic of the long Fordist period ^2^. To compete with others, companies started to produce more, leading to the introduction of new technologies and new models of work organization and management ^3^. To reduce labor costs, instead of typical employment, the productive restructuring of the 1980s was marked by the flexibilization of employment contract ^1^. In this system, the idea was to stimulate consumption, which, in turn, defined what would be produced, and not the opposite, as in series and mass production ^3^.

In this transformation, the standard employment relationship characterized by a full-time job for an indefinite period, with a fixed role, in a clearly defined company, in its premises, and with access to benefits and guarantees loses ground. Instead of stability, different employment bonds are highlighted, such as part-time, temporary, subcontracted, and outsourced work. These types of jobs are authorized by the State, in line with neoliberal ideas, in the form of regression and deregulation of labor rights acquired in previous decades ^4^.

The change in the paradigm of homogeneous and stable employment and its consequences on salaries, according to Castel ^5^, is related to a gradual increase in Brazil of people that has no basic survival conditions, including adolescents who wash cars, sell sweets, work in the sex market, and distribute and sell illicit drugs ^6^ ^,^ ^7^.

A large number of the workforce, on the one hand, has most occupations with the worst pay requiring lower educational or professional qualification, such as street vendors, delivery men, motorcycle couriers, security guards, general service providers, among other groups. On the other hand, on a small scale, cross-functional workers hold the highest paying jobs, responding under pressure to demands during the working days ^7^.

The significant growth in women’s participation in the labor market was accompanied by gender inequalities. Constraints arising from an asymmetrical sexual division of labor translate into salary difference between women and men in similar roles ^8^.

The spatial issue assumed new contours. Aided by technology, the boundaries between workplace, home, school, and leisure are blurred. Workers went out to the streets. Not only in the figurative sense of the term, but also literally. Currently, the streets are also places of work, with a large number of informal workers. In this new context, the topography of risk is different. Part of the workforce previously inserted in traditional factory environments, where most health incidents among workers occurred, is exposed to street risks. Today, almost three quarters of fatal work-related accidents in Brazil occur on the streets ^6^ ^,^ ^7^.

In the last century, when the industrialized economy predominated, salaried employees (indeed, the workforce was massively comprised of male workers) performed predefined operations in a fixed position, with a higher chance of being exposed to risks of accidents, deafness, and poisoning by chemical agents, among others. In this scenario, occupational hygiene and occupational medicine emerged. These two disciplines developed their own methods and techniques, reproducing the guiding conception of the Fordist work process. In Brazil, labor laws instituted health care in the company’s medical office and intervention criteria for fixed job positions. Based on this tradition, the occupational health paradigm was established ^9^.

Traditional risk agents and tolerance limits, as defined in the industrial era, despite their suitability for monitoring environments that maintain the characteristics from previous decades, are not sufficient to address the universe of working conditions in modified or recently created environments; for example, to monitor tension stemming from fast trips of motorcycle couriers. How to establish a tolerance limit, using measuring devices, for the perception of fear or anxiety in this type of situation? Under the pretext of promoting entrepreneurship and expanding job opportunities, ongoing job insecurity is accentuated in work managed by companies that control digital platforms. Thousands of workers are attracted to act as “their own entrepreneurs”, facing lack of protection at work, without guarantees of paid rest, vacations, accident insurance, etc. Fast pace and long working hours are the cause of health damage ^10^. What indices can be used to evaluate the environments where workers coordinated by digital platforms operate?

No previous change has brought such disintegration and dispersion of workers. It should be noted that factories, and later Fordist industries, were places of individual and collective integration and subjectivation, and places of socialization. In an extremely competitive environment, a feeling of permanent insecurity spreads, without bonds and acts of solidarity ^11^. Working without a feeling of belonging, people face the dispersion of their utopian energies. Hypotheses relate this idea to prevalent depressive suffering ^12^.

If traditional knowledge is not able to address emerging work activities, we must face the need to produce theoretical and methodological frameworks that are more suitable to the nature and magnitude of the problems already identified and others that are still poorly defined.

In this adverse scenario, the engagement of a significant number of young workers who, on the fringes of large Brazilian cities, work in the tenuous transition from licit to illicit, from legal to illegal, where risks are even more accentuated, is particularly worrying. Using clandestine water, electricity and cable TV connections, working with car dismantling and sale of such parts, sale of objects and goods without tax documents, distribution and marketing of psychoactive substances for illicit use, etc., are often the only option of revenue for young people discouraged by the lack of formal jobs. Involvement in these activities is a predictor of extreme vulnerability, indicating an increased risk of mental disorders and accidents associated with working in illegal markets ^13^.

Added to the list of concerns about unconsolidated research objects are the dimensions of racism, ableism, ageism, sexism, violence against queer people, among others, which are linked with job insecurity, justifying intersectional discussions. Labor analogous to slavery, which has been found here and there, is an example of another dimension, with situations of direct exploitation of transnational migrant women and children (working in prostitution in cities like Pacaraima, in Roraima State, or in small sewing factories in São Paulo) or rural workers (poor black people) recruited to be exploited in the large wine production in southern Brazil ^13^. Finally, changes in the sociodemographic and cultural profile of the workforce should also be highlighted.

The reality described above is an invitation to think about the directions of scientific investigation. If knowledge production methods have changed, if people are living with unprecedented effects of working conditions on health, then research objects and methods of doing science must also change. Reflecting on these and other health and work connections will require special efforts to address the epistemological challenges ^14^.

Union actions with a focus on health indicators have undoubtedly driven unquestionable advances. The reversal seen in the 1990s is a good example, when, with the consolidation of the Brazilian Unified National Health System (SUS), care and surveillance programs for workers’ health were created, of a public and universal nature ^15^. Developments like these continue to fuel academic-scientific investments that guide the creation of concepts and methods to address the problems related to workers’ health ^16^.

Researchers are improving knowledge about the inventiveness of individuals in work situations aiming to improve self-protection against fatigue, accidents, injuries, and illnesses. Investigations focused on the ethnographic aspects of work have reported relevant information. The genesis of accidents was postulated in interactions between production goals and work intensity ^17^. Studies on psychological processes underlying the work activity indicated that individuals are often prevented from developing self-protection strategies that were constructed through experience ^18^. Methods so far unprecedented in the course of research on health and work were used to analyze, for example, self-extermination events in the workplace ^19^.

Old processes and problems coexist with emerging ones. Advances and contradictions have transformed practices and generated knowledge that feeds current perspectives in the study of health and work relationships; for example, this reflection. However, this understanding still requires substantial innovations when considering the content of national magazines, which remain strongly anchored in the ideas of occupational health. To avoid producing “more of the same”, as often stated by scientific journal managers, incentives should be given to the academic-scientific community to replace the theoretical-methodological frameworks that proved to be powerful in the last century, but which are currently insufficient.

In 1962, Thomas Kuhn ^20^ argued that a paradigm shift is the driving force behind the production of scientific knowledge. In other words, science advances not because of knowledge accumulation, but because the scientific community is able to generate new approaches and concepts. For him, the dynamics of what he called the “scientific revolution” are motivated by academic commitments.

In view of the reality mentioned above, it may be useful to criticize old ways of conducting research that are insufficient nowadays, encouraging a movement that is based on experiences to promote a paradigm shift. For this reason, themes like uberization, activities on the streets or in illegal markets, among other forms of precarious work, should be incorporated into the research agenda.
